# Retention of the Native Epigenome in Purified Mammalian Chromatin

**DOI:** 10.1371/journal.pone.0133246

**Published:** 2015-08-06

**Authors:** Andreas H. Ehrensberger, Don-Marc Franchini, Philip East, Roger George, Nik Matthews, Sarah L. Maslen, Jesper Q. Svejstrup

**Affiliations:** 1 Mechanisms of Transcription Laboratory, Clare Hall Laboratories, The Francis Crick Insitute, London Research Institute, South Mimms, United Kingdom; 2 DNA Editing Lab, Clare Hall Laboratories, Cancer Research UK, London Research Institute, South Mimms, United Kingdom; 3 DNA Editing in Immunity and Epigenetics, IFOM-Fondazione Instituto FIRC di Oncologia Molecolare, Milano, Italy; 4 Bioinformatics and Biostatistics Group, The Francis Crick Insitute, London Research Institute, London, United Kingdom; 5 Protein Purification Facility, The Francis Crick Insitute, London Research Institute, London, United Kingdom; 6 Advanced Sequencing Facility, The Francis Crick Insitute, London Research Institute, London, United Kingdom; 7 MRC Laboratory of Molecular Biology, Francis Crick Avenue, Cambridge Biomedical Campus, Cambridge, United Kingdom; Southern Illinois University School of Medicine, UNITED STATES

## Abstract

A protocol is presented for the isolation of native mammalian chromatin as fibers of 25–250 nucleosomes under conditions that preserve the natural epigenetic signature. The material is composed almost exclusively of histones and DNA and conforms to the structure expected by electron microscopy. All sequences probed for were retained, indicating that the material is representative of the majority of the genome. DNA methylation marks and histone marks resembled the patterns observed in vivo. Importantly, nucleosome positions also remained largely unchanged, except on CpG islands, where nucleosomes were found to be unstable. The technical challenges of reconstituting biochemical reactions with native mammalian chromatin are discussed.

## Introduction

Eukaryotic DNA is stored as chromatin, a complex of DNA and protein in which 146 bp of DNA are wrapped around a core of histone proteins composed of two copies each of histones H2A, H2B, H3 and H4 [1]. Each such unit is known as a nucleosome. An additional ~15 bp between nucleosomes is protected by a single copy of histone H1. Even though the structural backbone of chromatin is simple, it acquires vast heterogeneity through the epigenome, consisting of post-translational modifications of histones, replacement of histones with histone variants, and covalent modification of individual base pairs on the DNA [2, 3]. New chemical features of chromatin continue to be discovered, both at the level of histone marks [4], DNA modifications [5], and subnucleosomal structures [6], thus making the complexity of the epigenome ever more apparent.

Biochemical studies on chromatin are typically performed using material reconstituted *in vitro* from naked DNA and free histone octamers [7]. The DNA used in such reconstitutions typically harbours artificial or semi-artificial sequences that favour the formation of nucleosomes, since natural sequences tend to disfavour nucleosome formation *in vitro*. In the cases where natural DNA was used for reconstitution [8, 9], histone marks and histone variants were either absent altogether or were scrambled randomly across the template. The additional layer of information contained in histone marks and variants has emerged as critical in all transactions involving chromatin, but is currently absent when chromatin is reconstituted *in vitro*. Even though advances have been made to incorporate histone marks [10, 11] and histone variants [12] into reconstituted nucleosomes, such material still lacks the combinatorial complexity of the natural material. Protocols in which individual loci are isolated as closed circles from yeast have been successful for biochemical reconstitutions, but are still limited to the purification of individual gene loci [13–15].

**Fig 1:**
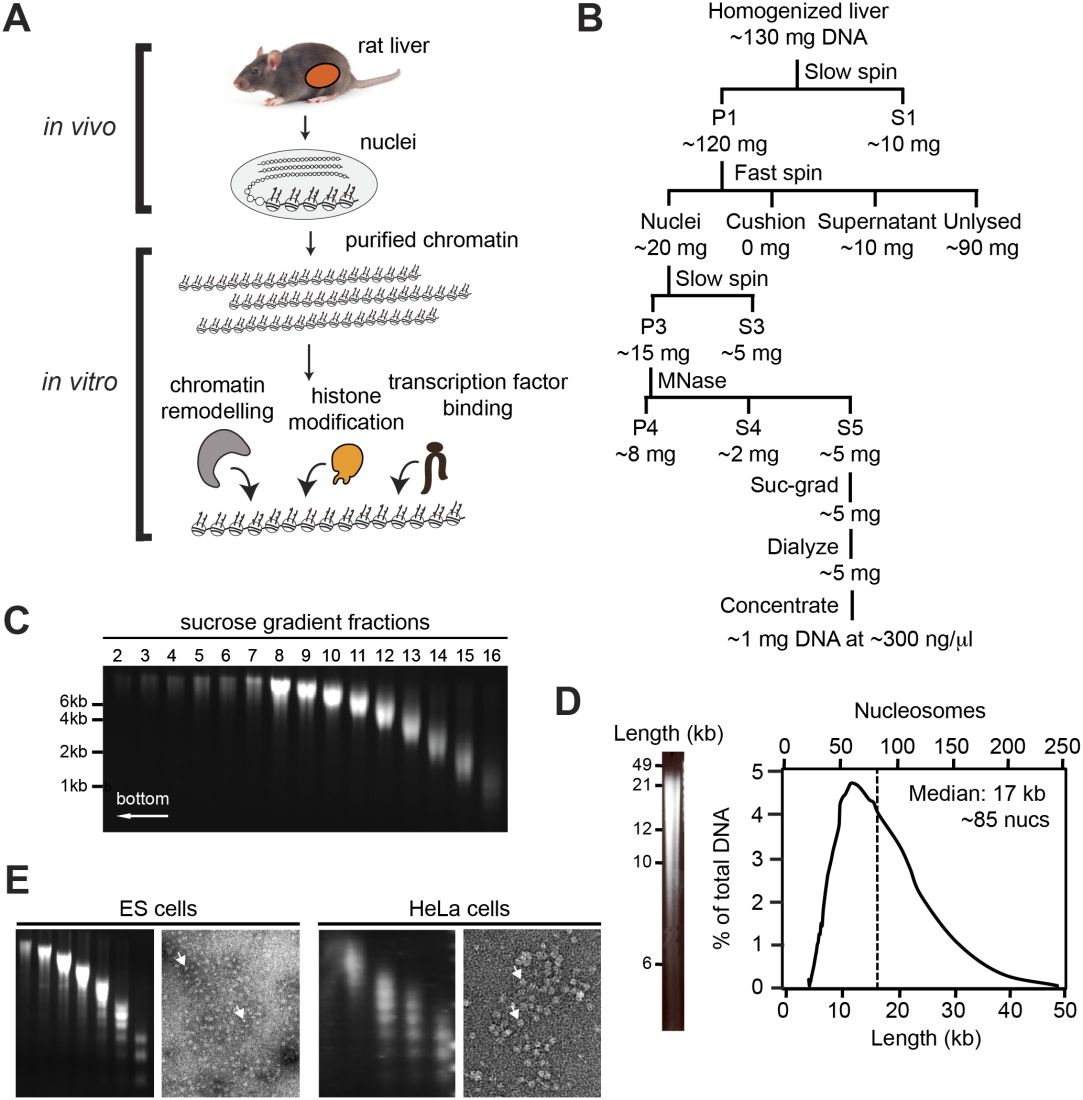
Purification procedure. (A) Diagram of chromatin purification and in vitro assays. Livers were removed from rats, and used to prepare nuclei. Chromatin was then extracted by digestion with MNase and centrifugation through a sucrose gradient. (B) Flowchart of purification procedure. The first then centrifugations serve to enrich nuclei, then follows digestion with MNase to solublize the chromatin, followed by sucrose gradient-centrifugation, dialysis and concentration. Amounts indicate approximate recovery of DNA. See Materials and Methods for description of individual fractions and steps. (C) Sucrose gradient. Agarose gel of DNA from sucrose gradient fractions. (D) Total length distribution. Agarose gel of pooled fractions, run as in C. Right panel shows total distribution of fragment lengths, calculated by normalizing the signal intensity to the fragment length. Top axis shows number of nucleosomes and bottom shows length in kilobases. Dashed line represents the mean fragment length. (E) Alternative sources of chromatin. Agarose gels of sucrose fractions and electron micrographs of total chromatin from fraction S5 of material prepared from mouse ES cells and HeLa cells. Arrows show individual nucleosomes.

Here, we describe a method for isolating chromatin from mammalian cells under conditions that retain the native set of DNA sequences, histone marks, nucleosome positions and DNA methylation patterns, and show that these features are largely retained. The protocol yields multi-kilobase-long fragments in which neighboring regulatory sequences are expected to stay connected after purification. This chromatin, termed genomic chromatin, is presented as a possible new substrate for the biochemical reconstitution of sequence-specific chromatin transactions (Fig 1A).

## Results and Discussion

### Isolation of genomic chromatin

Chromatin fragments were isolated from rat livers under gentle conditions that preserve their natural folding, by adapting the method of Kornberg *et al*. (1989) for the retention of epigenetic marks (Fig 1B). Rat livers were used because of their large size, the low heterogeneity of cell types [16], and their precedent as a source of chromatin [17].

In brief, nuclei were extracted from the livers through homogenization and ultracentrifugation into a sucrose cushion. Chromatin was solubilized by digestion with micrococcal nuclease (MNase) and fractionated by size over a sucrose gradient (Fig 1C). The relevant fractions were pooled, dialyzed and concentrated. Fig 1D shows an agarose gel and a trace of fragment lengths from a representative preparation. In this preparation, fragments had a median length of 17 kb, corresponding to about 85 nucleosomes. The final yields were ~1%, resulting in 1 mg of chromatin at ~300 ng/μl, starting from 5 rat livers. The greatest losses were incurred during homogenization of the tissue, where only 10–40% of nuclei could be released, and during MNase digestion, where ~50% of the chromatin remained insoluble and another ~25% was lost as mononucleosomes that leaked out of the MNase-treated nuclei. The protocol could be adapted to other cell types, including embryonic stem cells and HeLa cells (Fig 1E).

### Purity of genomic chromatin

**Fig 2:**
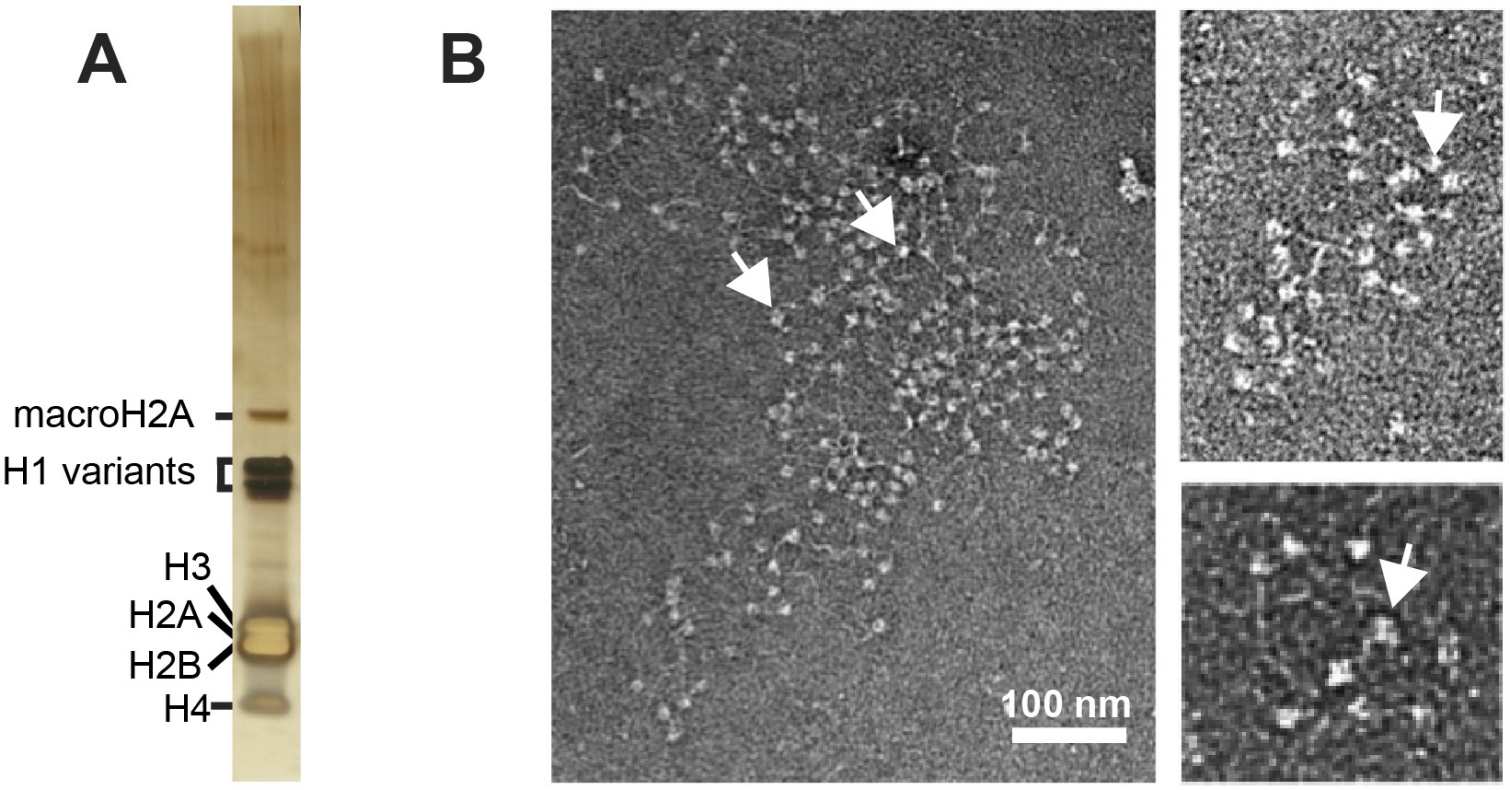
Purity of genomic chromatin. (A) Silver-stained 4–12% SDS-PAGE gel of genomic chromatin, with major bands highlighted. Note that all major bands are histones. (B) Electron micrographs of individual fragments of genomic chromatin from fraction S5 at three different magnifications. Arrows indicate individual nucleosomes.

When the material was analyzed by SDS-PAGE and stained with silver, the dominant bands were of the four core histones, of the histone variant macroH2A, and of several variants of histone H1 (Fig 2A). Mass spectrometry analysis of gel slices showed the presence of only histones and of three likely contaminants (hemoglobin, BSA and heterogeneous nuclear ribonucleoprotein M (Hnrpm)). A similar mass spectrometry analysis done in solution revealed the same proteins as found in the gel-slices, as well as minor traces of chromatin-binding proteins (Table 1). We were not surprised to find trace amounts of non-histone proteins still associated with the final material, given that the salt concentration throughout the purification was kept low to avoid nucleosome sliding. Nevertheless, quantitative analysis revealed a 1:1 ratio of protein to DNA, as would be expected for a chromatin preparation containing predominantly histones and DNA (data not shown). We note that the purpose of this protocol was to provide material that is pure enough for defined biochemical reconstitutions, but also as similar as possible to nuclear chromatin as it is found inside the living cell. Retention of structural integrity would thus invariably come with a cost of purity. While tightly-bound proteins would be expected to remain bound, their soluble counterparts will have been separated from the chromatin during sucrose gradient ultracentrifugation.

**Table 1:** Mass spectrometry analysis of genomic chromatin.

Hits from gel slices	Comments
H1.0	histone
H1.2	histone
H1a	histone
H1.3	histone
H3	histone
H2A.J	histone
H2A	histone
H2B	histone
H4	histone
macroH2A	histone
hemoglobin	likely secondary contaminant
BSA	likely secondary contaminant
Hnrpm	likely secondary contaminant
**Hits from solution**	
**Same proteins as from gel, and traces of:**	
HP1, topoisomerase, Cenpv, RNA helicase, Smarca5, SWI/SNF, MeCP2, PARP, others (less abundant)	

The purity and quality of the chromatin was further confirmed by electron microscopy, which showed chromatin fragments in the classical “beads-on-a-string” morphology of the 11 nm-fibre, with very few contaminants (Fig 2B). When individual nucleosomes could be counted on an electron micrograph, numbers per fiber corresponded well with those obtained by agarose length-analysis (compare with Fig 1D).

### Sequences are retained during purification

**Fig 3:**
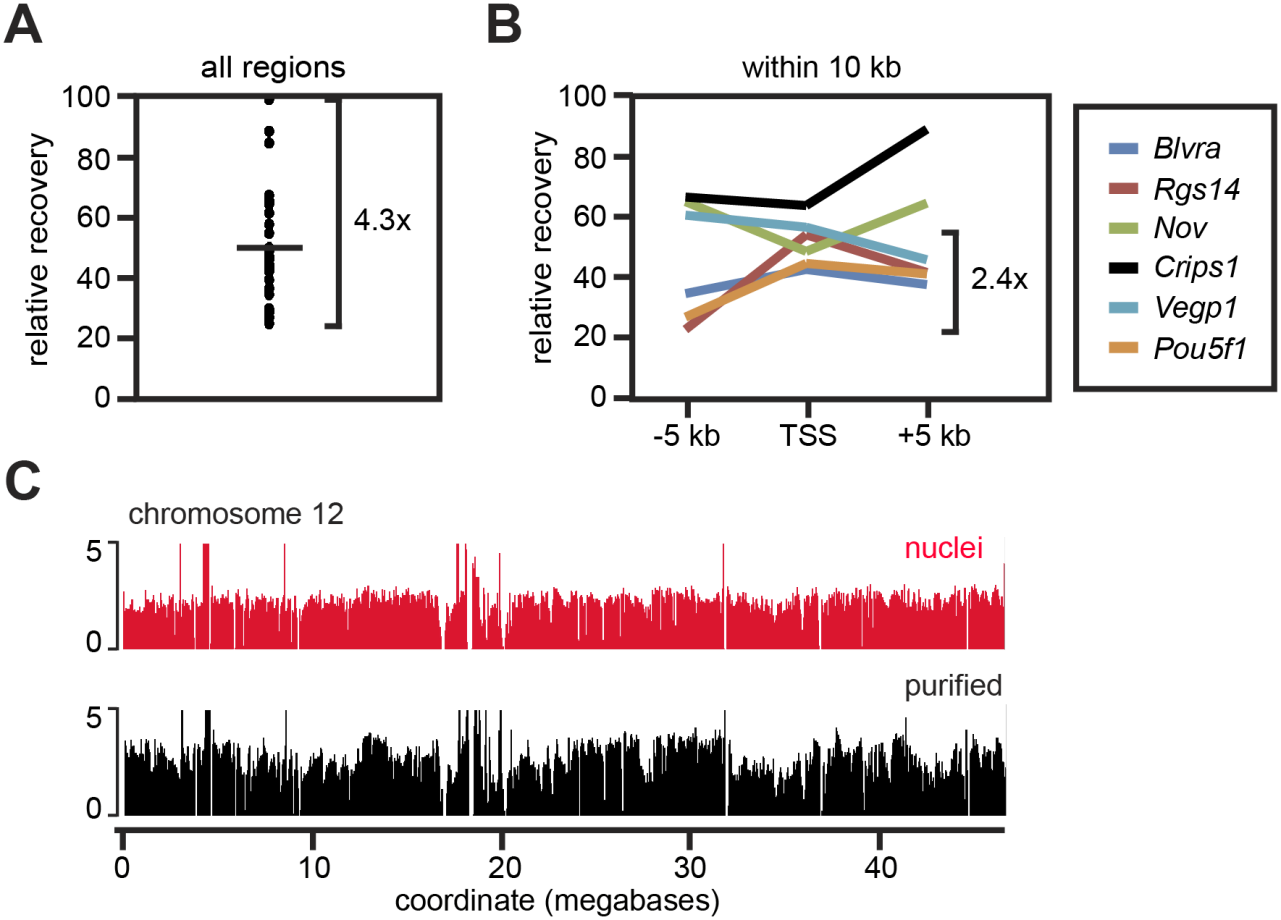
DNA sequence recovery. (A) Relative abundance of 29 genomic regions of ~100 bp, as quantified by qPCR relative to DNA in tissue. The difference in abundance between the most abundant and the least abundant sequence tested is indicated (4.3x). (B) Quantitation as in A, but using three primers within 10 kb for each of six genomic regions. The maximal difference in abundance between sites tested within 10 kb of the same chromosome is indicated (2.4x). (C) Distribution of sequences on chromosome 12, as obtained from paired-end sequencing of mononucleosomes prepared from nuclei or from purified genomic chromatin. Note that the sequences found in nuclei are also found in the purified material. See also S1 Fig.

We next asked how the recoveries of different sequences differed. We were initially expecting this not to be the case, since mammalian DNA exhibits a vast heterogeneity in degree of compaction [18], which might be reflected in different extraction efficiencies. When we compared the recoveries of 29 sequences of ~100 bp scattered across the genome, we found that they indeed differed, but never by more than 4.5-fold (Fig 3A). Within 10 kb of a single region, the recovery efficiencies differed by less than 2.5-fold (Fig 3B). Genomic sequencing of mononucleosomes prepared from the purified chromatin by secondary MNase digestion (S1 Fig) showed that sequences were indeed recovered across the genome, although with some variations between regions. A trace for chromosome 12 is shown as an example in Fig 3C. We conclude that this protocol is effective for extracting most, if not all, protein-coding genes in the form of native genomic chromatin.

### Histone mark levels and positioning are retained during purification

**Fig 4:**
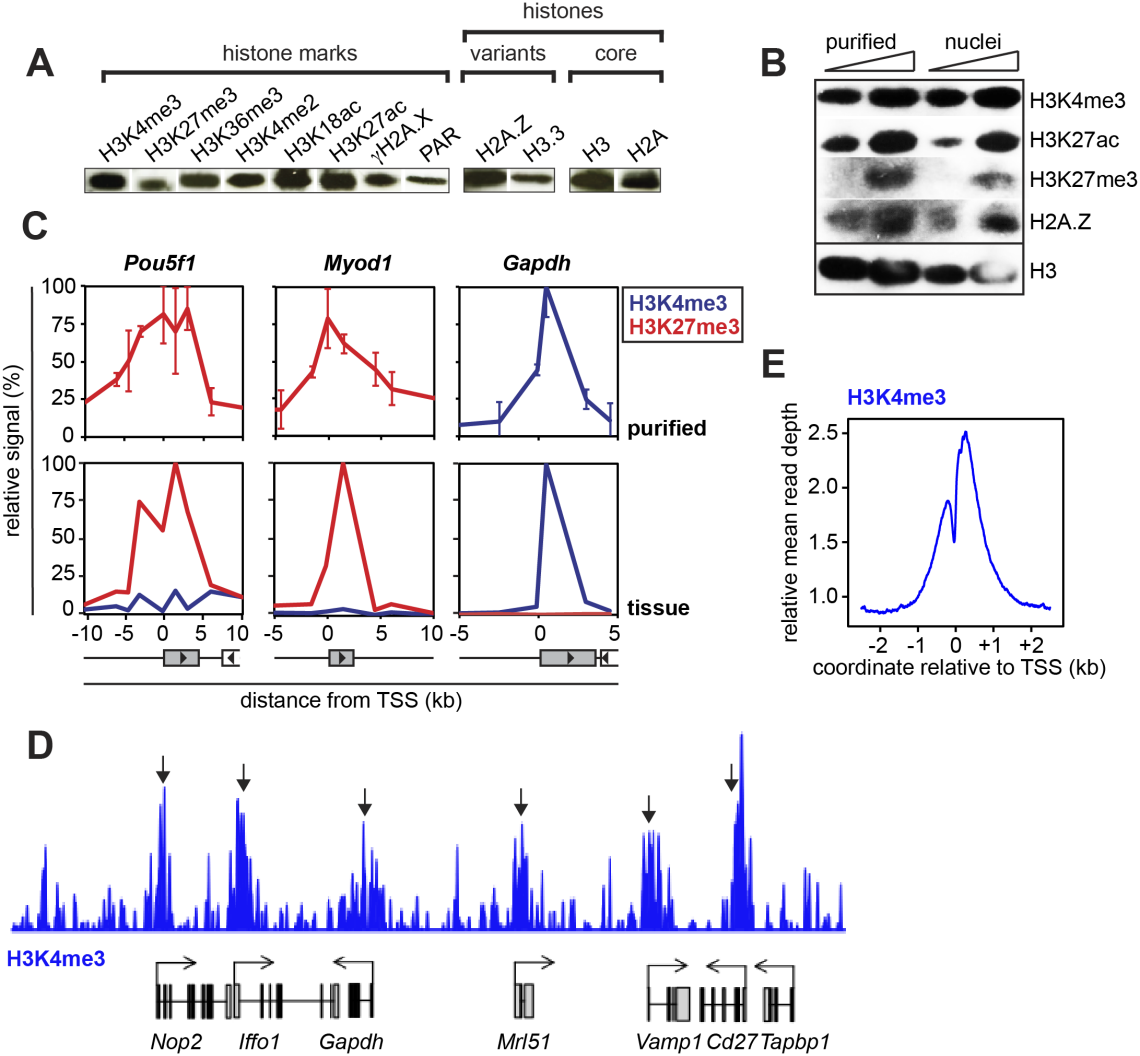
Histone mark retention. (A) Western blot of various histone marks, histone variants and core histones shows that all marks tested are detected after purification. For full lanes, see S2 Fig. (B) Comparative western blot shows similar levels of three histone marks and one histone variant in nuclei and in purified genomic chromatin relative to the levels of histone H3. (C) Chromatin IP of H3K4me3 and H3K27me3 in genomic chromatin and in tissue on three loci. Error bars show standard deviations from three biological replicates. Coordinate relative to TSS. (D) ChIP-Seq of H3K4me3 in genomic chromatin shows the expected pattern of peaks over active genes (arrows). (E) Metaprofile of D around the TSS shows the expected peak of H3K4me3 around the transcription start-site.

Histone marks help regulate biochemical processes on chromatin by recruiting key effector proteins or altering the stability of nucleosomes. Simple non-quantitative Western blotting for four methylation marks, two acetylation marks, one phosphorylation mark and poly-ADP-ribosylation (PAR) initially confirmed the presence of all the nine marks interrogated, as well as of the two histone variants probed for (Fig 4A). To get an idea about the relative amounts of several histone marks in the purified material relative to intact nuclei, we also compared signal intensities from purified chromatin with those from histones extracted directly from the nuclei (Fig 4B). The similar intensities of H3K4me3, H3K27me3, H3K27ac and H2A.Z suggest that purification does not result in large-scale demethylation or deacetylation, that H2A.Z is retained, and that proteolysis of the histone tails is minimal.

We next asked if the location of histone marks on genes is retained during purification, by comparing chromatin immunoprecipitation (ChIP) signal from crosslinked liver tissue with the patterns obtained by native ChIP (NChIP) of the purified genomic chromatin [19]. On all three genes tested, the pattern was largely retained (Fig 4C). ChIP-Seq of H3K4me3 on purified genomic chromatin confirmed the retention of the expected peaks over genes (Fig 4D) and in the genome-wide average (Fig 4E). We conclude that histone marks are retained in both abundance and location during the preparation of genomic chromatin.

### Nucleosome positioning of genomic chromatin

**Fig 5:**
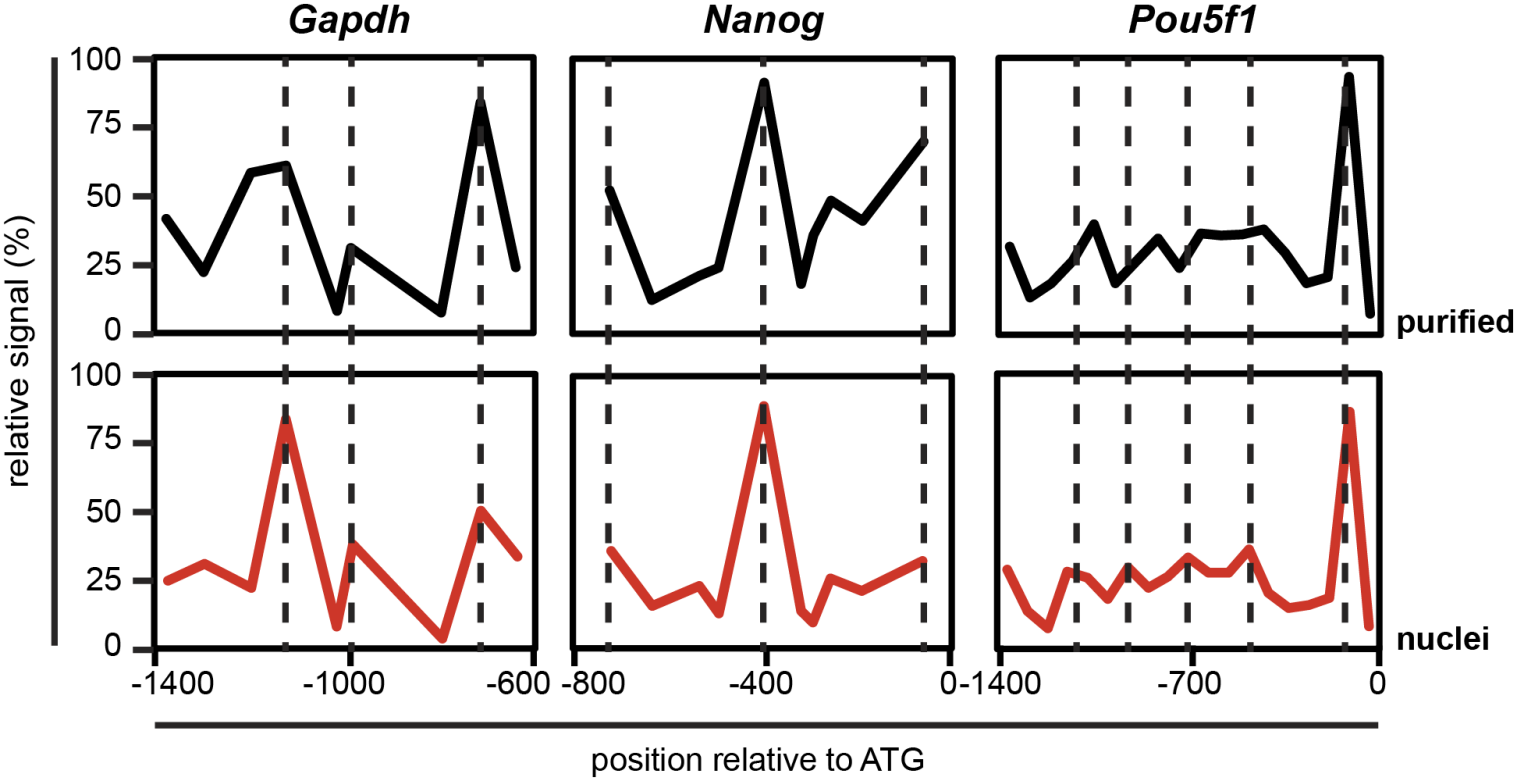
Nucleosome positions. Nucleosome positions on the promoters of one active locus (*Gapdh*) and two repressed loci (*Nanog* and *Pou5f1*) were mapped by ChIP-qPCR of mononucleosomes from purified genomic chromatin (black) and from nuclei (red). Peak height reflects nucleosome occupancy, and dashed lines denote the centers of nucleosome peaks in nuclei.

While the retention of peaks of histone marks shows that nucleosomes did not move significantly at the kilobases scale, it provides no indication about the extent to which individual nucleosomes slide or are displaced during genomic chromatin preparation. To assess the retention of nucleosome positions, we performed tiled quantitative PCR (qPCR) of mononucleosomal DNA, prepared from genomic chromatin, or from intact nuclei as a control (S1 Fig). At all three loci tested, the nucleosome organization remained largely unaffected after purification (Fig 5). This finding was both surprising and gratifying, since the process of purification is relatively long (~30 hours) and exposes the increasingly pure chromatin fragments to three different buffers. This suggests that DNA sequences remain largely stably bound as nucleosomes, even when extracted from their nuclear environment.

### DNA methylation remains intact during purification

One way in which cells silence genes is by methylating their DNA. This results in various repressive effects [20, 21], including recruitment of repressors [22], or additional compaction through binding of linker histone H1 [23].

**Fig 6:**
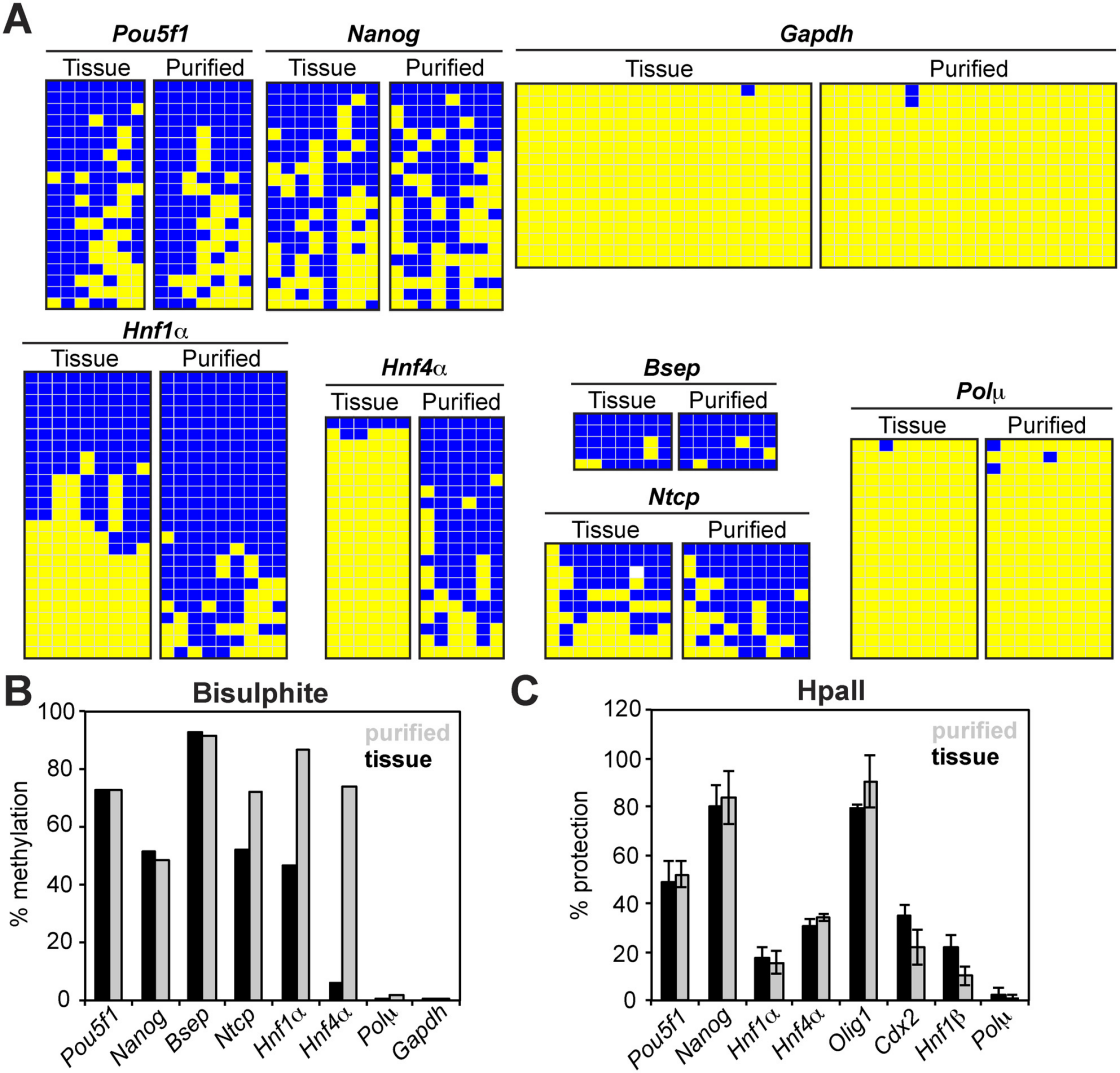
DNA methylation analysis. (A) Bisulphite sequencing analysis of genes from tissue and purified genomic chromatin. DNA was extracted (tissue) or purified as native genomic chromatin (purified) and subjected to bisulphite sequencing analysis. Yellow, blue and white boxes represent unmethylated, methylated and undetermined status of cytosine, respectively. (B) Quantification of methylation from bisulphite sequencing analysis performed in A. (C) *Hpa*II protection assay. DNA extracted from tissue or purified as native genomic chromatin was digested with methylation-sensitive restriction enzymes. Relative amount of amplified DNA from the *Hpa*II-treated sample was correlated to the relative amount of DNA amplified from the undigested sample, and expressed as percentage of protected DNA.

We evaluated the degree to which DNA methylation patterns are retained during purification by performing bisulphite sequencing analysis. As expected, the majority of loci retained their native methylation pattern (Fig 6A and 6B). Somewhat surprisingly, one locus, *Hnf4α*, stood out, with the methylated population appearing to be enriched during the purification. Nevertheless, DNA methylation analysis by digestion with the methylation-sensitive restriction enzyme HpaII confirmed the retention of the native pattern on all eight loci tested (Fig 6C). This time the methylation pattern of *Hnf4α* was detected as unchanged, suggesting a technical artefact with the first of the two detection techniques. We conclude that—as expected—most, if not all, loci retain their natural DNA methylation pattern during purification of genomic chromatin.

### CpG islands carry exceptionally unstable nucleosomes

**Fig 7:**
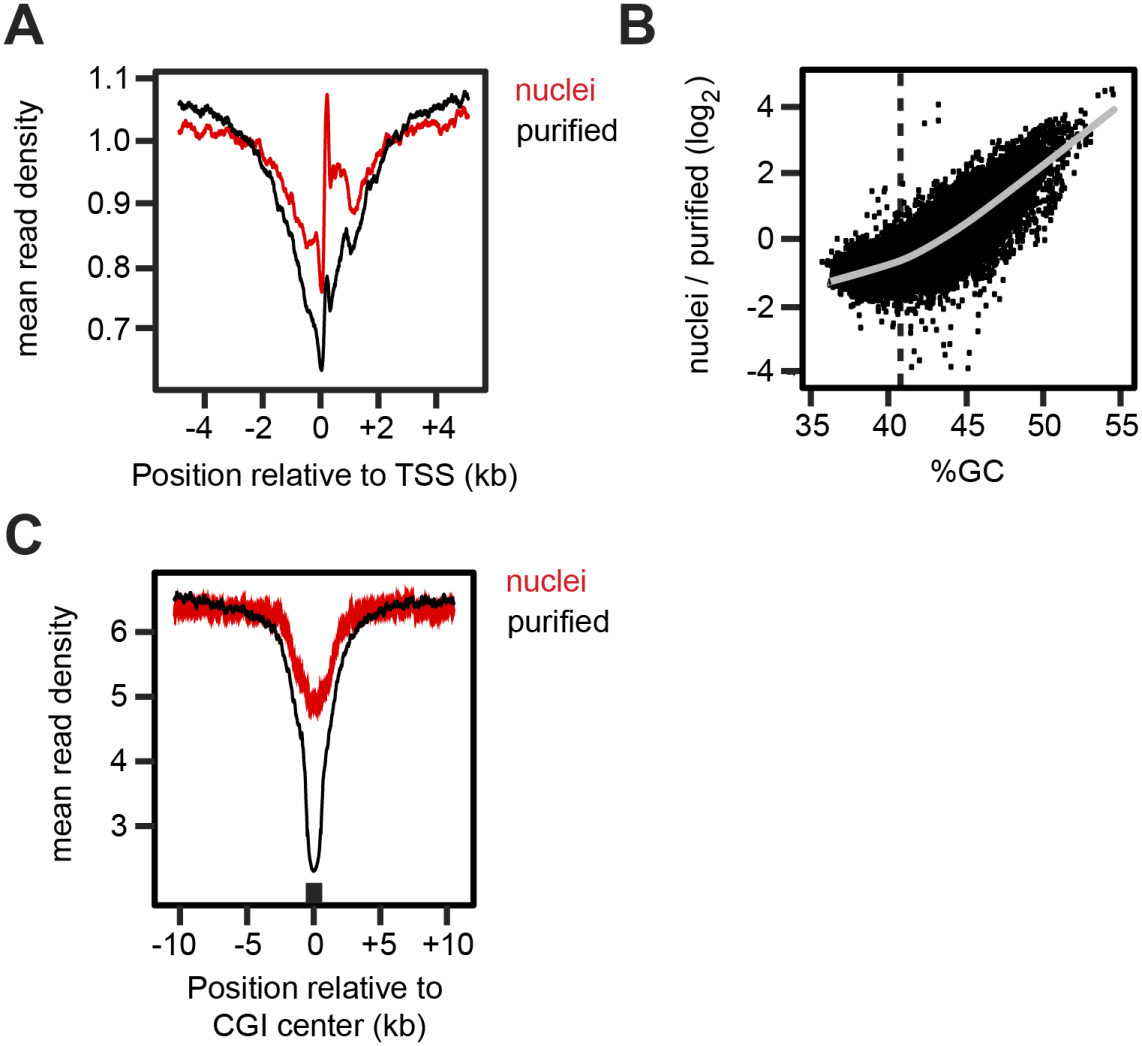
Loss of GC-rich nucleosomes during purification. (A) Normalized read counts for nucleosomes extracted from nuclei or from purified genomic chromatin digested to mononucleosomes, averaged around all TSS’s. Note greater depletion around the TSS in the purified material than in the nuclei. (B) Nucleosome loss after purification as a function of nucleosomal GC content. Nucleosome read counts from nuclei and purified chromatin were counted in 500 bp windows across the genome. The log2 ratio of the two is displayed. The grey line shows a fitted Loess function, and the dashed line shows the average genomic GC content of 41%. (C) Normalized read counts averaged around all CpG islands (CGI).

When mononucleosomes (S1 Fig) derived from purified genomic chromatin fragments were subject to next-generation sequencing, we noticed that in the genome-wide average, the signal was significantly lower on promoters than on other genomic regions. A dip in read counts in the region from -2 kb to +2 kb of the transcription start site (TSS) was apparent in the purified chromatin compared to mononucleosomes prepared from nuclei (Fig 7A). When we investigated the reason for this difference, we found that nucleosome loss had been particularly pronounced on regions with higher GC-content, with a roughly linear relationship between histone loss in the purified material and GC-content (Fig 7B). It seems reasonable to speculate that this selective loss of nucleosomes is due to CpG-islands, which are enriched in 70% of mammalian promoters [24] and have previously been found to exhibit a reduced ability to form nucleosomes *in vitro* [25]. When the metaprofile of reads was aligned around all CpG islands, we found this indeed to be the case (Fig 7C). We conclude that a subset of promoters lose nucleosomes in the CpG islands surrounding the TSS during purification. In a separate study, we found that yeast promoters also harbour exceptionally unstable nucleosomes, although in that case their low stability correlates with poly(dA:dT) tracts, rather than CpG islands (Ehrensberger *et al*., manuscript in preparation). This difference is most likely explained by the different features of yeast and mammalian genomes, with yeast promoters being characterized by poly(dA:dT) tracts [26], while mammalian promoters often harbour CpG islands [24]. The low stability of nucleosomes of high GC-content was previously reported for reconstituted nucleosomes and might facilitate constitutive transcription of housekeeping genes *in vivo* [25].

### Applications and technical challenges

**Fig 8:**
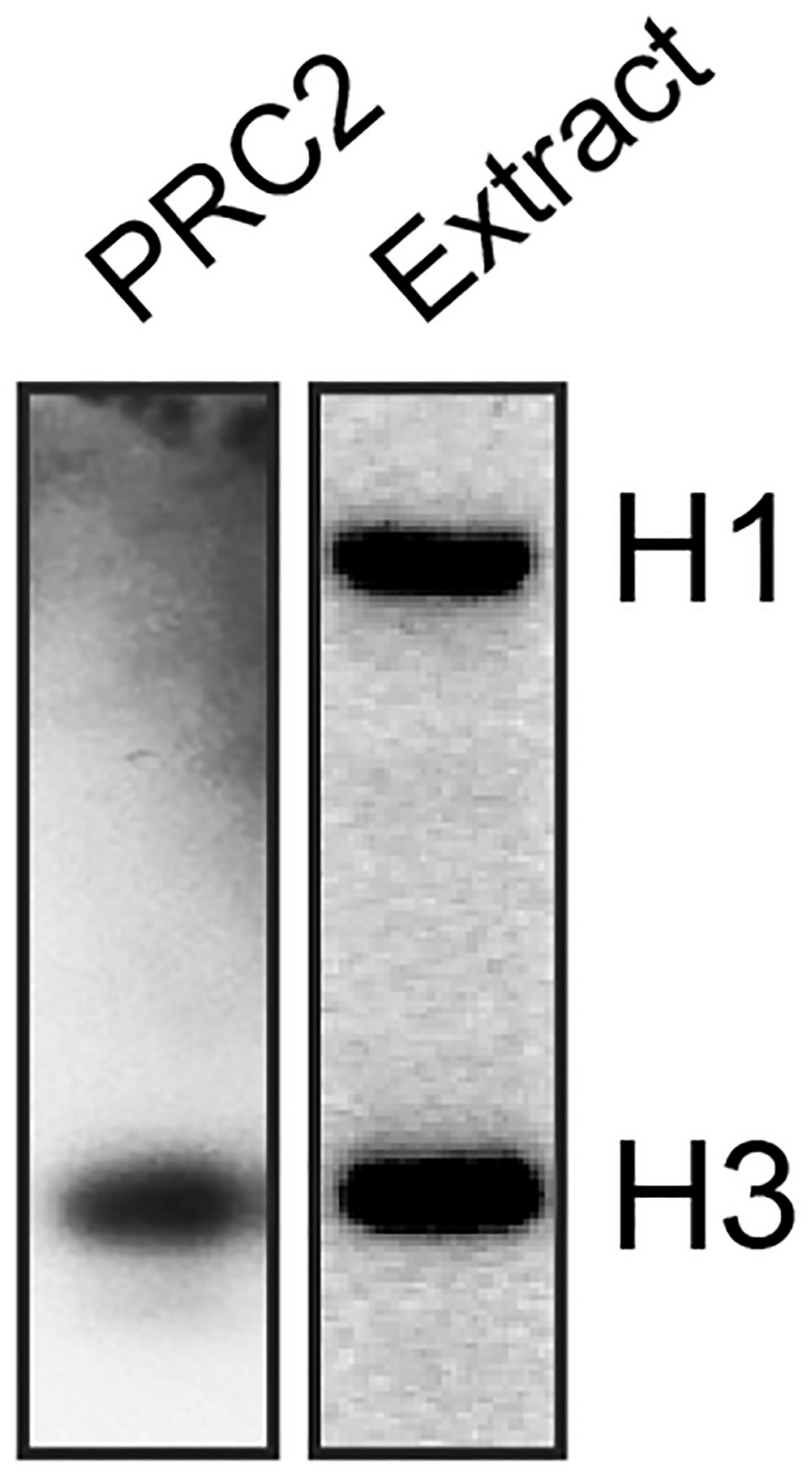
Methylation of genomic chromatin by PRC2 and extract. Chromatin was incubated with PRC2 or whole-cell extract, and ^3^H-SAM, and the product run on an SDS-PAGE gel for analysis by fluorography. PRC2 methylates its known substrate, histone H3, whereas extract methylates both histones H3 and H1.

The original purpose of this purification protocol was to use genomic chromatin for reconstituting locus-specific events on chromatin. Methylation reconstituted *in vitro* indeed showed that ^3^H could be incorporated into chromatin incubated in the presence of ^3^H-*S*-adenosylmethionine and the methyltransferase PRC2 or whole-cell extract (Fig 8). However, several attempts to detect locus-specific histone methylation events by native *in vitro* ChIP remained unsuccessful, presumably due to the high background of natural methylation marks and the vast complexity of the substrate (data not shown). In addition, the physical properties of the purified material appeared to render it highly challenging for biochemical studies: (1) chemical crosslinking using formaldehyde, as would be required for protein binding studies, made the chromatin stick to beads non-specifically (S3A Fig), and (2) a general propensity of the material to aggregate was also observed, as reflected in the inability of even mono-nucleosomes to enter native gels (S3B and S3C Fig), and in the precipitation of the full fragments in even low concentrations of magnesium ions (S3D Fig). A candidate culprit for the thus far sub-optimal behaviour of genomic chromatin during biochemical manipulations is the linker histone H1, which is typically absent from preparations of recombinant nucleosomes. Histone H1 plays a central role in chromatin compaction [27], where it is required for the formation of higher-order structures. Even as monomers, H1-containing nucleosomes have been reported to aggregate in solution [28].

Two solutions might help render genomic chromatin suitable for biochemical manipulation by reducing its stickyness and propensity to aggregate: (1) concentrations of divalent cations might be adjusted to levels that are still high enough for enzymatic function, but also low enough to prevent aggregation (see S3D Fig), and (2) histone H1 might be stripped from the chromatin altogether through exposure to a cation-exchange resin [27]. The material might then be used to study the locus-specificity of transcription factors and enzymes and how this correlates with epigenetic marks and sequence features. For example, does a given transcription factor prefer to bind to regions that are rich in H3K4me3, or does PRC2 prefer to methylate nucleosomes that are on promoters? Such studies would provide an advance over current approaches with reconstituted chromatin, which lacks the epigenetic complexity that might be required to establish the locus-specificity of transcription factors and chromatin-modifying enzymes.

The present study shows that chromatin can be isolated to a high degree of purity under conditions that preserve the majority of its natural epigenetic marks. The technical challenges that we faced could largely be attributed to a high propensity of the material to aggregate and stick to beads or to other proteins. Despite these difficulties, we are hopeful that in the future this protocol can be modified further to render the chromatin more suitable for the reconstitution of genome-wide chromatin transactions in fields as diverse as transcription, replication and DNA repair.

## Materials and Methods

### Preparation of chromatin from rat livers

The protocol for preparing rat liver chromatin was adapted from Kornberg et al. (1989) with an added sucrose gradient, a concentration and dialysis step, and with conditions modified for the preservation of epigenetic marks. Live rats were received from purchase in the morning and killed within 20–30 minutes of arrival by a technician trained in methods of humane killing using “exposure to carbon dioxide gas in a rising concentration”, in accordance with the Code of Practice for the Humane Killing of Animals under Schedule 1 to the Animals (Scientific Procedures) Act (ASPA) of 1986, stated as “appropriate for rodents, rabbits and birds up to 1.5 kg”. There was no need for review by an Institutional Animal Care and Use Committee, since the animals were not housed, fed, or used for experiments on live animals. The alternative of purchasing frozen livers was tested (purchased from Harlan Laboratories UK), but they were found to result in very low yields of chromatin, in addition to the danger that repeated freeze-thawing posed for the structure of native chromatin.

Salt concentrations throughout the purification were kept low enough to minimize nucleosome sliding, and the entire purification was performed at 4°C, as rapidly as possible, and in the presence of deacetylase inhibitors (trichostatin A and sodium butyrate) and protease inhibitors. In the final Chromatin Dialysis Buffer, the concentrations of monovalent and divalent salts were kept such that the chromatin remained folded but not aggregated (based on [29]).

All buffers contained 0.15 μM spermine, 0.5 μM spermidine, 0.15 mM β-mercaptoethanol, 5 mM sodium butyrate, 5 nM trichostatin A, 3 mM PMSF, 2 mM benzamidine, 2 μM leupeptin and 1 μg/ml Pepstatin A. Five fresh, unfrozen, rat livers were cooled on ice, minced and resuspended in a small volume of Buffer A (12% sucrose, 15 mM NaCl, 60 mM KCl, 15 mM Tris pH 7.5, 2 mM EDTA, 0.5 mM EGTA). They were pulverized in five strokes using a motor-driven Potter-Elvehjem tissue homogenizer, then layered over 5 ml 1:1 mix of Buffer A and Buffer B (72% sucrose, 15 mM NaCl, 60 mM KCl, 15 mM Tris pH 7.5, 0.1 mM EDTA, 0.1 mM EGTA) in a JA-21 tube. After centrifuging for 15 minutes at 10,000 rpm in a JA-21 rotor, the supernatant (fraction “S1”) was decanted and the pellets resuspended in 2 ml Buffer B for each gram of liver tissue (fraction “P1”). The resuspended pellets were layered over a cushion of 4 x 9 ml Buffer B in SW32 tubes and centrifuged for 90 minutes at 27,000 rpm in a SW32 rotor. The nuclei that entered the cushion contained 10–40% of the total chromatin. The rest was mostly left behind inside the cells that could not be homogenized and remained floating on top of the solution (see Fig 1A for yields from every step). The nuclei were resuspended in 5 ml Buffer C (12% sucrose, 15 mM NaCl, 60 mM KCl, 15 mM Tris pH 7.5), transferred to SW41 tubes, and centrifuged at 5,000 rpm for 5 minutes in a SW41 rotor for further purification (fractions “P3” and “S3”). The pellet containing the nuclei was resuspended in 100 ul Buffer C per gram of liver tissue, distributed into 100–200 μl aliquots, frozen in liquid nitrogen and stored at -80°C.

For micrococcal nuclease (MNase, New England Biolabs) digestion of purified nuclei *in situ*, the optimal concentration for obtaining fragments of the desired length was first optimized in small-scale trials. For the large-scale preparation, a large number of aliquots were pooled (eg. 20 x 200 μl = 4 ml). They were preheated for 2 minutes at 37°C, before adding 20 mM CaCl_2_ and MNase at the desired concentration (eg. 1.25 units/μl in one preparation). The digestion time was kept short, usually to less than two minutes, in order to minimize nucleosome sliding. The digestion was stopped with 10 mM EDTA. Samples were placed on ice for a few minutes, then distributed into eppendorf tubes and centrifuged for 1 minute at 18,000 x g. The supernatant containing the shortest fragments (“S4”) was discarded, and the pellet resuspended in 900 μl Buffer D (10 mM Tris pH 7.5, 1 mM EDTA, 1 mM EGTA). The low salt expands and solubilizes chromatin fragments, thereby separating them from the insoluble nuclei. After centrifuging 5 minutes at 18,000 x g, the supernatant (“S5”) was loaded on the sucrose gradient and the pellet (“P5”) discarded. About half of the chromatin remained insoluble, despite various attempts to optimize the extraction procedure. The sucrose gradient was prepared in SW41 tubes by layering solutions containing 45, 40, 35, 30, 25 and 20% sucrose in 30 mM NaCl, 10 mM Tris pH 7.5, 1 mM EDTA, 1 mM EGTA. S5 was layered on top of the gradient and centrifuged for 3.5 hours at 41,000 rpm in the SW41 rotor. Fractions of 500 μl were collected by piercing the bottom of each tube with a 23-gauge needle. A quick agarose gel analysis was used to determine what fractions contained the fragments of the desired length. These were then pooled and dialyzed overnight in Chromatin Dialysis Buffer (30 mM NaCl, 10 mM Tris pH 7.5, 1 mM EDTA, 1.5 mM MgCl_2_, 10% glycerol) to remove the sucrose using a 12.4 kDa MWC dialysis bag (Sigma). The next morning, the ~13 ml of material was concentrated to ~2 ml by covering the bag in Aquacide II polymer, which extracts water due to its high hygroscopicity. After 5–10 hours, the chromatin was dialyzed a second time for 2.5 hours, before aliquoting into 100 μl fractions, flash-freezing in liquid nitrogren and storing at -80°C. Final yields from 5 rat livers were in the order of 1 mg at a concentration of 0.3 mg/ml (chromatin concentration is given by the concentration of the DNA).

### Preparation of chromatin from cells grown in tissue culture

Mouse ES cells of line 46C [30] were obtained as a kind gift from Dr. Matthieu Gerard (CEA Saclay). Chromatin from cells grown in tissue culture (HeLa, mouse ES cells) was prepared in a manner similar to rat liver chromatin, except that nuclei were enriched under milder conditions due to the absence of connective tissue. All buffers contained protease and deacetylase inhibitors as listed above. HeLa or ES cells were grown and harvested for a cell pellet of 20–25 ml. The pellet was washed with cold PBS, then resuspended in 25 ml CR-B-0.34 (50 mM potassium acetate pH 7.9, 10 mM Tris pH 7.5, 3 mM CaCl_2_, 2 mM magnesium acetate, 0.1 mM EDTA, 0.1% NP-40, 0.1 mM BME; and 0.34 M sucrose). Resuspended cells were homogenized 35 times using a loose pestle and a manual glass homogenizer. After centrifuging for 20 minutes at 3,000 rpm in a JA-25.5 rotor, nuclei were resuspended in 30 ml CR-B-0.34 without NP-40, then centrifuged for 20 minutes at 3,000 rpm (JA-25.50 rotor). The enriched nuclei were resuspended in 25 ml CR-B-0.34 without NP-40, then layered on 2 x 10 ml 1:1 CR-B-0.34: CR-B-2.1, then centrifuged for 15 minutes at 10,000 rpm (JA-25.50 rotor). The pellets were resuspended to a total volume of 45 ml in CRB-2.1, then layered on 2 x 19 ml CR-B-2.1 in SW32 tubes, and centrifuged for 90 minutes at 27,000 rpm in the SW32 rotor. Pellets were resuspended to 16 ml CR-B-0.34, then centrifuged for 30 minutes at 30,000 rpm in SW41 tubes, and lastly resuspended to 5 ml CR-B-0.34 and aliquoted to 20 x 250 μl tubes for flash-freezing and storage at -80°C. MNase digestion and subsequent steps were performed as for rat livers.

### DNA sequence quantitation

The relative abundance of sequences in genomic chromatin were quantified by qPCR relative to the input DNA extracted from liver tissue.

### Native chromatin immunoprecipitation for histone marks from genomic chromatin

100 μl of genomic chromatin (30 μg) were adjusted to 5 mM CaCl_2_, before MNase (400 U) digestion was performed for 2 minutes at 37°C. The reaction was stopped with 20 mM EGTA and by placing it on ice. The optimal digestion time and MNase concentration were optimized for each preparation. After digestion, 900 μl IP-Buffer (150 mM NaCl, 50 mM Tris pH 8.0, 2 mM EDTA, 0.1% NP-40, 0.01% SDS, 0.1 mg/ml BSA, 0.1 mg/ml herring sperm DNA and protease inhibitors) was added, and the chromatin aliquot to 6 x 150 μl for immunoprecipitation. It was then incubated for 4.5 hours at 4°C with 25 μl Protein A-Dynabeads preloaded with 5 ml antibody, and rinsed with the same buffer. Beads were then washed three times with IP-Buffer. The buffer conditions had to be kept mild, since chromatin had not been crosslinked. In trial experiments we found that formaldehyde crosslinking aggregated the chromatin and rendered it unsuitable for immunoprecipitation. After washing, the DNA was eluted with 50 μl Elution Buffer (1% SDS, 50 mM Tris pH 7.5, 50 mM NaCl, 5 mM EDTA) for 20 minutes at room temperature. 100 μl TE pH 8.0 were added to the eluate, which was then purified DNA using a Qiagen PCR purification kit. The same antibodies and quantitation procedure were used as for tissue ChIP.

### Chromatin immunoprecipitation for histone marks from tissue

Preparation of chromatin: 80 mg of frozen liver tissue was minced, rinsed with PBS, and resuspended in 500 μl 700 mM Hepes pH 7.8 with 12% formaldehyde. The tissue was incubated for 10 minutes at room temperature for crosslinking, quenched with 350 μl 2 M glycine, incubated 5 minutes at room temperature and washed three times with cold PBS. For sonication, it was resuspended in 1.6 ml ChIP Lysis B (50 mM Tris pH 8.0, 10 mM EDTA, 1% SDS, protease inhibitors), aliquoted to 6 x 200 μl, sonicated 2 x 5 minutes with 30 seconds on 30 seconds off at max power on Bioruptor on ice. The sheared chromatin was centrifuged for 5 minutes as 18,000 x g, pooled and aliquoted into 50 ml fractions, flash-frozen and stored at -80°C. Immunoprecipitation: 12 aliquots containing 600 μl chromatin were diluted 1:5 in ChIP-Buffer (150 mM NaCl, 50 mM Tris pH 8.0, 2 mM EDTA, 1% Triton X-100, 0.01% SDS, 0.1 mg/ml BSA, 0.1 mg/ml herring sperm DNA and protease inhibitors). The immunoprecipitation was performed by standard procedures using antibodies Abcam 8580 (H3K4me3) and 6002 (H3K27me3), and in-house rabbit IgG with Protein A Dynabeads (Life Technologies). Washes were performed once each with ChIP-Buffer, HSB (500 mM NaCl, 50 mM Tris pH 8.0, 2 mM EDTA, 1% Triton X-100, 0.1% SDS) and LiCl-B (250 mM LiCl, 10 mM Tris pH 8.0, 2 mM EDTA, 1% deoxycholate, 1% NP-40). After eluting with Elution Buffer (1% SDS, 100 mM NaHCO_3_), the DNA was purified with a Qiagen PCR purification kit. Quantitative PCR was performed using a Bio-Rad CFX-96 thermocycler.

### Nucleosome positioning analysis

Mononucleosomal DNA from nuclei: to one 100 μl aliquot of nuclei in Chromatin Dialysis Buffer were added 3 mM CaCl_2_ and 1,200 U MNase. After incubation for 1 minute at 37°C, the reaction was stopped with 50 mM EGTA, and the DNA purified with the Qiagen PCR purification kit. Mononucleosomal DNA was gel-purified from an agarose gel using the Qiagen Gel purification kit.

Mononucleosomal DNA from genomic chromatin: to one 100 μl aliquot of chromatin were added 900 μl Chromatin Dialysis Buffer and CaCl_2_ was adjusted to 5 mM. The chromatin was preheated for 1 minutes to 37°C, before adding 1,000 U MNase and digesting for 2 minutes at 37°C. Digestions were stopped with 25 mM EGTA, then processed like mononucleosomal DNA from nuclei. Nucleosome positioning analysis: as input, we used undigested DNA either from nuclei or from genomic chromatin. The amount of each sequence in mononucleosomal DNA was then compared to the amount in the input DNA using qPCR.

### Whole-cell extract for histone methylation

The protocol was adapted from [31] using 46C embryonic stem cells.

### Histone methylation assay for gel analysis

1–5 μg genomic chromatin was incubated with 60 μg ESWX-298 or 770 ng PRC2 and 0.5 μCi 3H-*S*-adenosylmethionine (13 mM, 1 μl) in 50 mM Tris pH 8.0, 5 mM MgCl_2_ and 4 mM DTT for 1 hour at 30°C. The reaction was stopped by adding SDS loading buffer, boiled for 5 minutes and the resulting species separated on a 4–12% NuPAGE gel (Invitrogen). Analysis was performed by standard fluorography enhanced with Amplify (GE Healthcare).

### Crosslinking and stickiness study of genomic chromatin

The original purpose of this procedure was to bind transcription factors to chromatin in order to assess their location preference across the genome. In the process of setting up the protocol, we found that even in the absence of added transcription factors chromatin binds to antibody-bound magnetic beads. This was investigated further: to 11 μl genomic chromatin (7 μg), were added 14 μl Chromatin Dialysis Buffer and 10 μl CHD-B100 (100 mM NaCl, 50 mM Tris pH 7.5, 10 mM EDTA, 0.1% NP-40, 10% glycerol). The mixture was incubated for 5 minutes at room temperature, before adding 10 μl 5x FX-Buffer (250 mM potassium acetate 7.6, 25 mM MgCl_2_, 50 mM Hepes pH 7.9), 1 μl 5 mM NTPs and 5 μl ESX-Buffer (100 mM KCl, 25 mM Hepes pH 7.9, 12 mM MgCl_2_, 1 mM EDTA, 20% glycerol, 2 mM DTT). After incubation for 20 minutes at room temperature, formaldehyde was added at 0.1% or 1%, and the mixture incubated for 10 minutes at room temperature, stopped with glycine and incubated for 5 minutes at room temperature. For digestion, 12 mM CaCl_2_ and 80,00 U MNase was added, before incubating for 5 minutes at 37°C and stopping the digestion with 80 mM EGTA. 400 μl IP-Buffer was added and the mixture incubated for 2 hours at 4°C with 25 μl Protein A Dynabeads bound with rabbit anti-FLAG antibody (Sigma F7524). The bead-chromatin mixture was washed three times with 1 ml IP-Buffer, before analyzing the beads for DNA and protein content.

### Native nucleosome gels

Mononucleosomes were prepared from genomic chromatin by secondary digestion with micrococcal nuclease or reconstituted *in vitro* as described ([32]. They were loaded on a 2% agarose gel and run at 60 V, or on a 5% PAGE gel and run at 100 V. Both gels were run in 0.5x TBE at 4°C. Gels were subsequently stained with ethidium bromide.

### Preparation of recombinant PRC2

Generation of infective virus particles: Competent E.coli DH10 Bac cells were transformed with plasmids coding for each member of the PRC2 complex (kindly provided by Nicola Thomä, Friedrich Miescher Institute). White colonies were picked from freshly transformed plates and grown in liquid culture in order to purify bacmid DNA. This DNA was then used to generate virus by transfecting Sf21 insect cells. Two further rounds of amplification generated high titre virus stocks suitable for infecting large scale cultures for expression of the complex.

Expression of PRC2 complex in Sf21 cells: A 500 ml culture of Sf21 insect cells at a density of 1 x 10^6^ cells/ ml was infected with viruses encoding each subunit of the PRC2 complex (Eed, Rbbp4, Suz12 and Ezh2) at an MOI of 2. Successful infection of the cells, was determined by assessing the cell density and diameter 24 hours post infection. The infected culture was allowed to grow for a total of three days at 27°C with constant shaking (110 rpm), after which time the cells were harvested and stored at -80°C.

Purification of the PRC2 complex: Cells were lysed in buffer containing 50 mM Tris pH8.0, 250 mM NaCl, 10% glycerol, 0.5% Triton X-100, 1 mM EDTA, 1 mM NaF, 1 mM Na_2_VO_4_, 10 mM B-glycerophosphate, 1 mM DTT and protease inhibitors and then sonicated to ensure complete lysis. The insoluble fraction was removed by centrifugation (21,000 rpm for 15 minutes at 4°C) and the soluble fraction incubated with 200 μl bed volume of NiNTA at 4°C for 1 hour. The resin was washed extensively with PBS, 1 mM DTT, 10 mm imidazole and the complex eluted in 3 x 2 ml fractions with PBS pH 7.2 containing 200 mM imidazole. The elutions were combined and concentrated to 0.6 ml and further purified by applying the complex to a Superose 6 size exclusion column equlilibriated with PBS pH 7.2, 5% glycerol, 1 mM DTT. Fractions containing the PRC2 complex were pooled and concentrated to 200 μg/ml and snap frozen as 20 μl aliquots until required.

### Mononucleosome reconstitution

Recombinant mononucleosomes were assembled on the Widom 601 sequence [33] by the method of [32].

### DNA Methylation Assays

CpG methylation status of the genomic DNA and purified chromatin was monitored by bisulphite sequencing with the EZ DNA Methylation-Gold kit (Zymo Research). Bisulphite-treated DNA was amplified by the use of a nested PCR strategy with the Zymo Taq PreMix (Zymo Research) and the corresponding pair of primers for each genes analyzed (see S1 Table). PCR products were separated on 1% agarose gels, purified by Qiaquick Gel Purification (Qiagen), cloned into carrier plasmids by using the TOPO TA Cloning Kit (Invitrogen) and individual clones were sequenced. Data were analyzed using the BISMA software [34].

*Hpa*II assay protocol was performed by digesting the DNA either with the *Hpa*II or *Msp*I restriction enzymes. Digested and undigested DNA were analyzed by real-time quantitative PCR on a LightCycler 480 Real-Time PCR System (Roche Applied Sciences) using LightCycler 480 SYBR Green I master mix (Roche Applied Sciences) and specific primers (see S1 Table). The relative amount of amplified DNA was measured by threshold cycle amplification (Ct). The amplification fold change was calculated using the ΔΔCt method between the *Hpa*II and the undigested samples, each correlated with the *Msp*I-treated sample, and was expressed as percentage of protected DNA (i.e. percentage of methylation at the CCGG site).

### Computational analysis

Illumina paired-end reads were aligned to the rat genome (rn4) using BWA. After filtering, correctly mapped pairs were extended and merged with their corresponding pairs. Fragment sizes within the range 140–200 bp were selected for and duplicate fragments removed. Fragments mapping to regions with continuous coverage of greater than 1,000 basepairs were also discarded. This gave 42,159,565 fragments for “purified” and 32,623,888 for “nuclei”. Each sample was scaled to 50 x 10^9^ fragments prior to further analysis.

### Electron microscopy

5 μl of chromatin dialyzed overnight against water were added to a carbon-coated, glow-discharged, 3 mm Transmission Electron Microscope (TEM) grid and left to absorb for 1 minute. The grid was stained with 2% uranyl acetate in deionized H2O, blotted from the side with filter paper, and left to air-dry for 30 minutes at room temperature. The grids were imaged in an 120 kV G2 Spirit Twin transmission electron microscope (FEI Company, Eindhoven) with an Ultrascan CCD camera (Gatan Inc, Pleasanton).

### Primers

All primer sequences are provided in S1 Table.

## Supporting Information

S1 Fig Mononucleosomal DNA. DNA extracted from mononucleosomes prepared by secondary digestion with MNase (agarose gel analysis).
(TIF)

S2 Fig Western blot of histone marks. Samples probed for for Fig 4A, but showing full lanes and available marker bands. RLC = rat liver chromatin (genomic chromatin), CT = calf thymus histone (Sigma # H9250). Bands used for Fig 4A are marked with red circles.
(TIF)

S3 Fig Stickyness and aggregation of genomic chromatin. A. Stickiness of genomic chromatin after crosslinking. Chromatin was crosslinked with formaldehyde, the reaction stopped with glycine, and the chromatin incubated with antibody-bound beads (the FLAG epitope, against which the antibody was raised, is absent in the chromatin). After washing, beads were probed for histone H3 by Western blotting, or for DNA by crosslink-reversal, phenol-chloroform extraction and agarose gel analysis. Note that both H3 and DNA are present on beads after crosslinking, indicating non-specific binding. B and C. Native electrophoresis of mononucleosomes. Nucleosomes were prepared by secondary digestion of purified genomic chromatin, or through *in vitro* reconstiution, and then run on a 2% agarose gel (B) or on a native 4–20% polyacrylamide gel (C) at 4°C. Note that genomic, but not recombinant, nucleosomes remain in the well, indicating aggregation. The two asterisks show DNA that was lost from the recombinant nucleosomes. (D) Aggregation of chromatin fragments with magnesium. Purified chromatin was incubated with varying concentrations of magnesium chloride and centrifuged at 18,000 x g for one minute. The DNA concentration of the precipitated portion was quantified from both the pellet and the supernatant.
(TIF)

S1 Table Primer sequences. (XLSX)
