# Correction: Retention of the Native Epigenome in Purified Mammalian Chromatin

**DOI:** 10.1371/journal.pone.0141250

**Published:** 2015-10-16

**Authors:** Andreas H. Ehrensberger, Don-Marc Franchini, Philip East, Roger George, Nik Matthews, Sarah L. Maslen, Jesper Q. Svejstrup

There are errors in the Funding section. The correct funding information is as follows: Funding was provided by Cancer Research UK, http://www.cancerresearchuk.org/, European Research Council grant number 268797, and an EMBO Long-Term Fellowship to AHE.
