# Supplementary figures and images for: Retention of the Native Epigenome in Purified Mammalian Chromatin
Source: PLoS One. 2015 Aug 6;10(8):e0133246. doi: 10.1371/journal.pone.0133246 (PMC4527833; doi:10.1371/journal.pone.0133246)

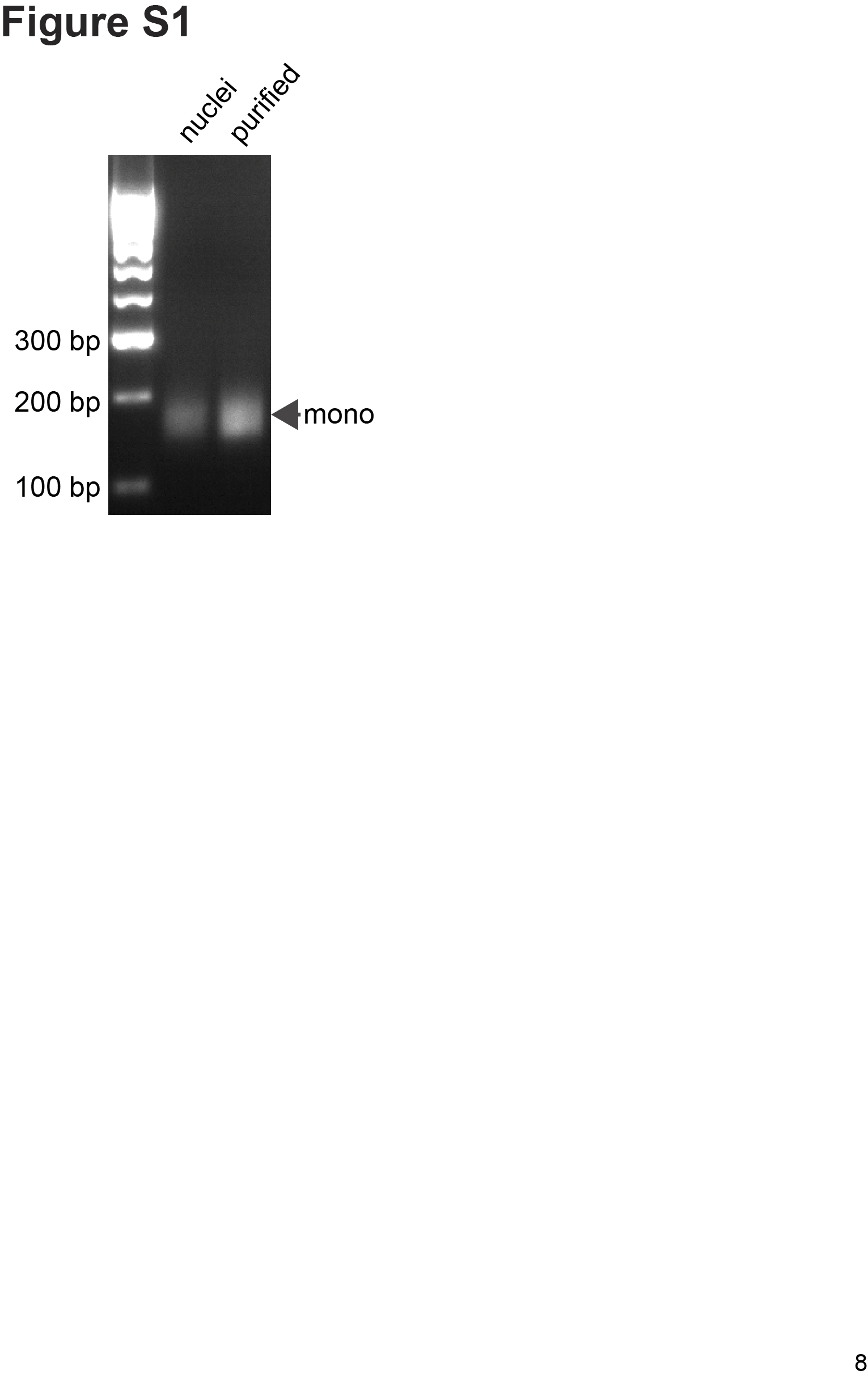

Supplement: S1 Fig — DNA extracted from mononucleosomes prepared by secondary digestion with MNase (agarose gel analysis). (TIF) [file pone.0133246.s001.tif]

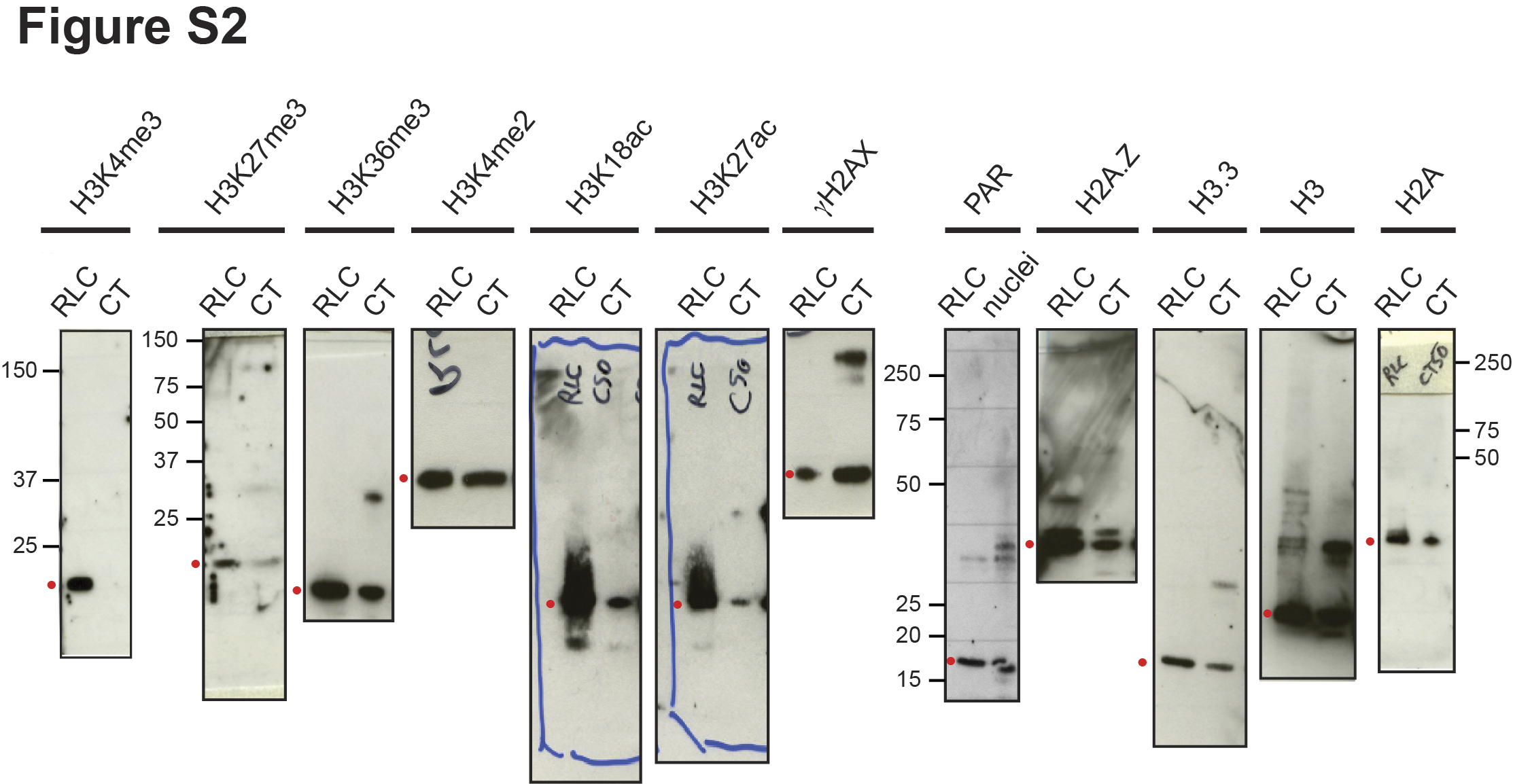

Supplement: S2 Fig — Samples probed for for Fig 4A, but showing full lanes and available marker bands. RLC = rat liver chromatin (genomic chromatin), CT = calf thymus histone (Sigma # H9250). Bands used for Fig 4A are marked with red circles. (TIF) [file pone.0133246.s002.tif]

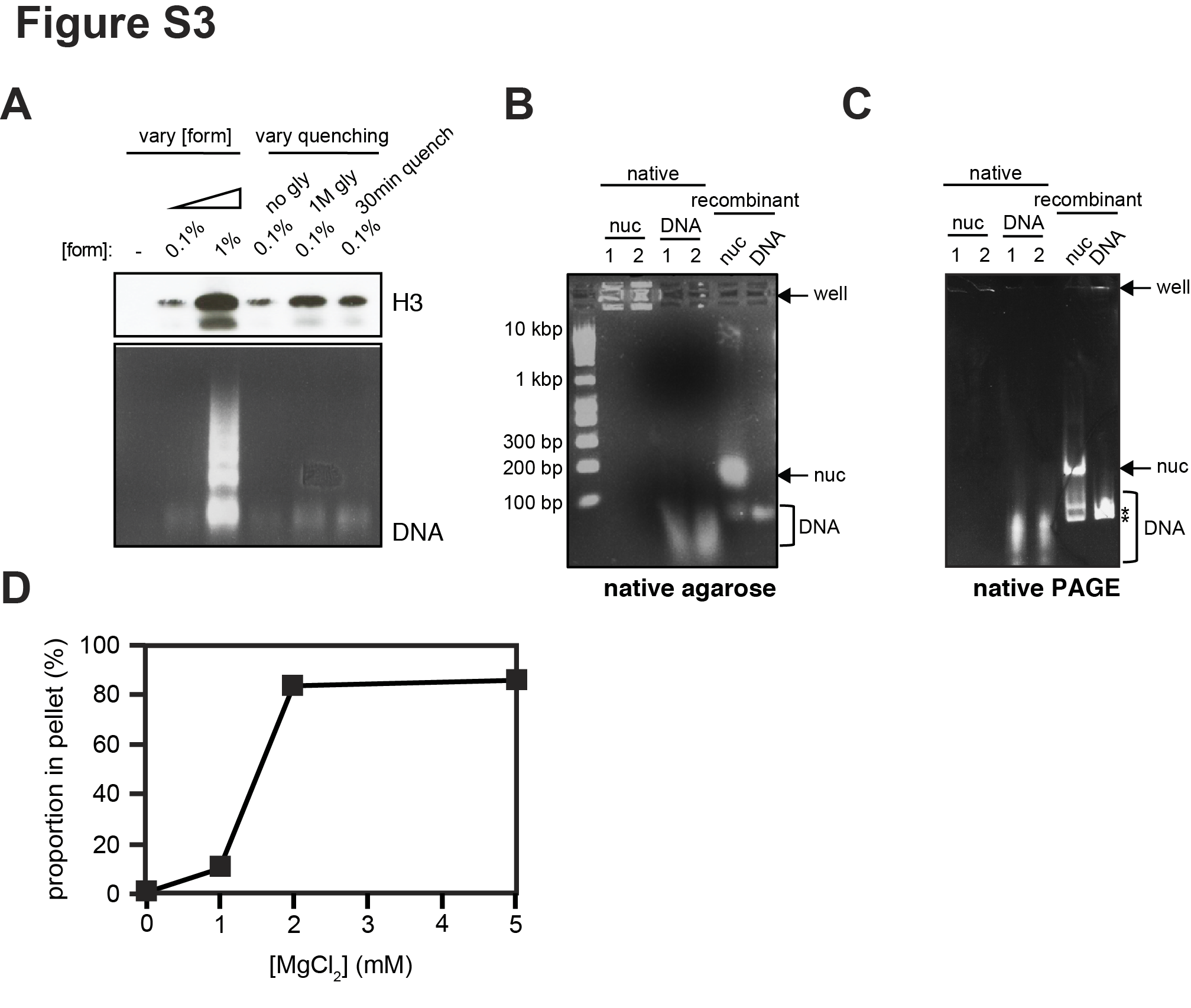

Supplement: S3 Fig — A. Stickiness of genomic chromatin after crosslinking. Chromatin was crosslinked with formaldehyde, the reaction stopped with glycine, and the chromatin incubated with antibody-bound beads (the FLAG epitope, against which the antibody was raised, is absent in the chromatin). After washing, beads were probed for histone H3 by Western blotting, or for DNA by crosslink-reversal, phenol-chloroform extraction and agarose gel analysis. Note that both H3 and DNA are present on beads after crosslinking, indicating non-specific binding. B and C. Native electrophoresis of mononucleosomes. Nucleosomes were prepared by secondary digestion of purified genomic chromatin, or through in vitro reconstiution, and then run on a 2% agarose gel (B) or on a native 4–20% polyacrylamide gel (C) at 4°C. Note that genomic, but not recombinant, nucleosomes remain in the well, indicating aggregation. The two asterisks show DNA that was lost from the recombinant nucleosomes. (D) Aggregation of chromatin fragments with magnesium. Purified chromatin was incubated with varying concentrations of magnesium chloride and centrifuged at 18,000 x g for one minute. The DNA concentration of the precipitated portion was quantified from both the pellet and the supernatant. (TIF) [file pone.0133246.s003.tif]
